# Fatal elective DDD pacemaker implantation

**DOI:** 10.1007/s12471-017-1049-1

**Published:** 2017-10-30

**Authors:** B. Klop, L. J. P. M. van Woerkens, M. Bijl

**Affiliations:** Department of Cardiology, Albert Schweitzer Hospital, Dordrecht, The Netherlands

## Answer

**Fig. 1:**
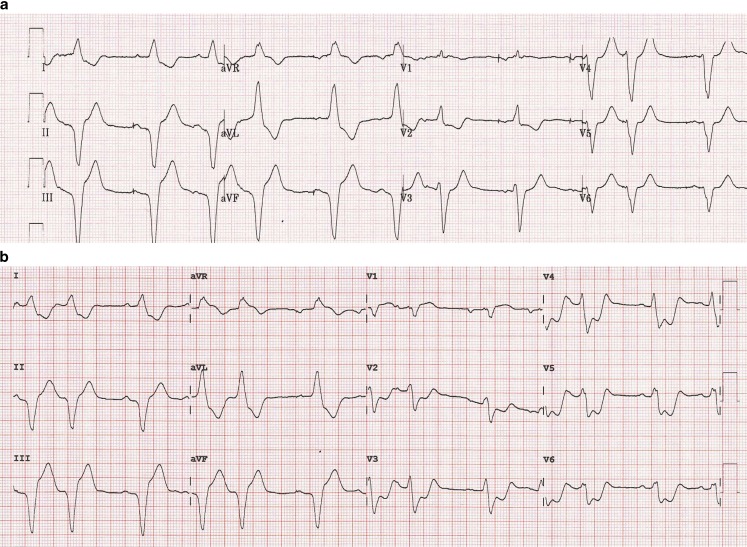
**a** The initial electrocardiogram obtained directly after implantation of the DDD pacemaker, **b** The repeat electrocardiogram

The initial electrocardiogram obtained immediately after implantation of the dual chamber (DDD) pacemaker showed normal atrioventricular pacing with a premature atrial complex after every two beats and an expected left bundle branch block pattern (LBBB) (Fig. 1a). The repeat electrocardiogram showed a similar rhythm with an LBBB, but with concordant ST segment elevation in lead aVR and concordant ST segment depression in leads V2 to V6 (Fig. 1b). The patient developed a refractory cardiogenic shock and a coronary angiography was obtained, which revealed extensive three-vessel disease without thrombi or acute occlusions. Fluoroscopy did not show any signs of lead displacement. The patient died during emergency percutaneous coronary intervention.

The modified Sgarbossa criteria are specific for cardiac ischaemia in patients with ventricular pacing and LBBB: concordant ST segment elevation in any lead, concordant ST segment depression in leads V1 to V3 and discordant ST segment elevation >5 mm in any lead or a ratio of ST segment elevation to S‑wave amplitude of 0.25 or more [1–3].

The patient was asymptomatic before implantation of the pacemaker. The low heart rate probably protected him against myocardial ischaemia. After inducing ventricular pacing, the heart rate was normalised to 70 beats per minute, which resulted in panischaemia with subsequent refractory cardiogenic shock due to extensive three-vessel disease. In retrospect, early intervention with lowering of the basal pacing rate might have reversed the development of ischaemia and subsequent cardiogenic shock.
